# Adrenocortical carcinoma in a West White Terrier: clinical and diagnostic approach

**DOI:** 10.29374/2527-2179.bjvm005424

**Published:** 2024-10-11

**Authors:** Fernanda de Paula Sesti, Gabriel Marchiori Gonzaga, Flávia Maria Tavares Manoel Zimmer, Tatiane Viana de Souza Cruz, Bruno Alberigi

**Affiliations:** 1 Veterinarian, Programa de Pós-graduação em Medicina Veterinária (PPGMV), Departamento de Clínica e Cirurgia Veterinária (DMCV), Instituto de Veterinária (IV), Universidade Federal Rural do Rio de Janeiro (UFRRJ). Seropédica, RJ. Brazil.; 2 Veterinarian, MSc, Autonomus, Rio de Janeiro, RJ, Brazil; 3 Veterinarian, Autonomus, Volta Redonda, RJ, Brazil; 4 Veterinarian, DSc. DMCV, IV, UFRRJ. Seropédica, RJ, Brazil.

**Keywords:** endocrinology, elastography, ultrasonography, endocrinologia, elastografia, ultrassonografia

## Abstract

Adrenocortical carcinoma (ACC) is a rare condition in dogs. This type of tumor can be functional, hormone-producing, or nonfunctional. Bilateral adrenal tumors are uncommon, whereas unilateral adrenal tumors are more prevalent. Abdominal ultrasonography is crucial for detecting these lesions, which are characterized by heterogeneous parenchymal characteristics. Furthermore, elastography, a technique that assesses tissue stiffness, is useful for differentiating between malignant and benign lesions. This paper describes a case of ACC in a West Highland white terrier. The dog initially presented with dermatological changes and increased adrenal tissue stiffness, as detected by elastography. After additional examinations, including computed tomography, an adrenalectomy was successfully performed. Histopathological analysis confirmed the diagnosis of ACC. Post-surgical follow-ups included periodic ultrasonography to monitor the remaining adrenal gland. A nodular lesion was identified in the left adrenal gland during the follow-up, highlighting the importance of continuous monitoring. This case underscores the significance of an integrated approach, from the initial evaluation to the post-treatment follow-up for the effective management of ACC in dogs.

## Resumo

O carcinoma adrenocortical (ACC) é uma condição rara em cães. Esse tipo de tumor pode ser funcional, produtor de hormônios ou não funcional. Tumores adrenais bilaterais são incomuns, enquanto tumores adrenais unilaterais são mais prevalentes. A ultrassonografia abdominal é crucial para detectar essas lesões, que são caracterizadas por características parenquimatosas heterogêneas. Além disso, a elastografia, uma técnica que avalia a rigidez do tecido, é útil para diferenciar entre lesões malignas e benignas. Este artigo descreve um caso de ACC em um West Highland White Terrier. O cão apresentou inicialmente alterações dermatológicas e aumento da rigidez do tecido adrenal, conforme detectado pela elastografia. Após exames adicionais, incluindo tomografia computadorizada, uma adrenalectomia foi realizada com sucesso. A análise histopatológica confirmou o diagnóstico de ACC. Os acompanhamentos pós-cirúrgicos incluíram ultrassonografia periódica para monitorar a glândula adrenal remanescente. Uma lesão nodular foi identificada na glândula adrenal esquerda durante o acompanhamento, destacando a importância do monitoramento contínuo. Este caso ressalta a importância de uma abordagem integrada, desde a avaliação inicial até o acompanhamento pós-tratamento para o manejo eficaz do ACC em cães.

## Introduction

Approximately 1–2% of dogs are affected by adrenal tumors, which can be classified into functional and non-functional malignancies that either produce or do not produce hormones, respectively (Lunn & Boston, 2020). Adrenocortical carcinomas (ACCs) or adenomas are the predominant neoformations originating from the adrenal cortex and have the capacity to secrete androgen hormones, aldosterone and cortisol (Machida et al., 2008).

Bilateral adrenal tumors rarely affect dogs, with their unilateral involvement being more common (Cook et al., 2014). In addition, metastases occur in 7–50% of cases, primarily affecting the liver, lungs, kidneys, and mesenteric lymph nodes (Labelle et al., 2004). Another possible complication is the invasion of the caudal vena cava by adrenal tumors owing to their anatomical proximity. In a study conducted in 2020, Lunn reported that 50% of patients with ACC may exhibit such an invasion. Neoformations can be detected using abdominal ultrasonography. These lesions are characterized by heterogeneous parenchymal characteristic, a round shape, and the presence of nodular areas (Melián et al., 2021). Lesions measuring ≥2 cm or evidence of vascular invasion may suggest malignancy (Cook et al., 2014).

In conjunction with ultrasound, elastography can be used to analyze tissue stiffness through mechanical excitation (Holdsworth et al., 2014). Tissue elasticity is assessed by the force of the applied pressure and degree of deformation (Li & Snedeker, 2011).

Feliciano et al. (2018) compared elastography findings with those of the acoustic impulse radiation method and histopathology in breast carcinomas. They found that elastography can help identify complex carcinomas with moderate accuracy.

In human medicine, elastography is used to evaluate the adrenal glands and is effective for identifying adrenal neoformations and characterizing malignant and benign lesions (Słapa et al., 2014).

In cases of adrenal neoformation, computed tomography (CT) is necessary to delimit neoplastic lesions, assess the presence of vascular invasion in three dimensions, and aid in surgical planning for the resection of the affected adrenal gland (Bennaim et al., 2019).

The therapeutic approach for patients with ACCs can be based on the use of medication to control clinical signs; however, surgical removal remains the preferred treatment to avoid invasion into adjacent structures and prevent metastasis (Massari et al., 2011).

Adrenalectomy can be performed using minimally invasive techniques such as laparoscopy in cases of small-to-moderate adrenal neoformations (Balsa & Culp, 2019). In cases of vascular invasion, conventional laparoscopy is indicated because of the need to perform a cavotomy and remove possible thrombi (Mayhew et al., 2019).

Histopathological examination is essential for the diagnosis of ACC and should be performed after surgical removal (Renaudin et al., 2018). The Utrecht score is calculated using histopathological data and the Ki67 gene proliferation index to obtain a more accurate prognosis and provide information on therapeutic targets (Sanders et al., 2019).

This report presents the clinical case of a 10-year-old female West Highland white terrier who presented with a neoformation in her right adrenal gland, dermatological changes, and increased tissue rigidity in the adrenal glands on elastographic analysis.

## Case report

The dog in this report was monitored due to recurrent skin changes, skin peeling, hyperpigmentation mainly in the abdomen and axillary regions, and recurrent otitis. Since the dermatopathy did not respond to treatment, an associated underlying endocrinopathy was suspected.

**Figure 1:**
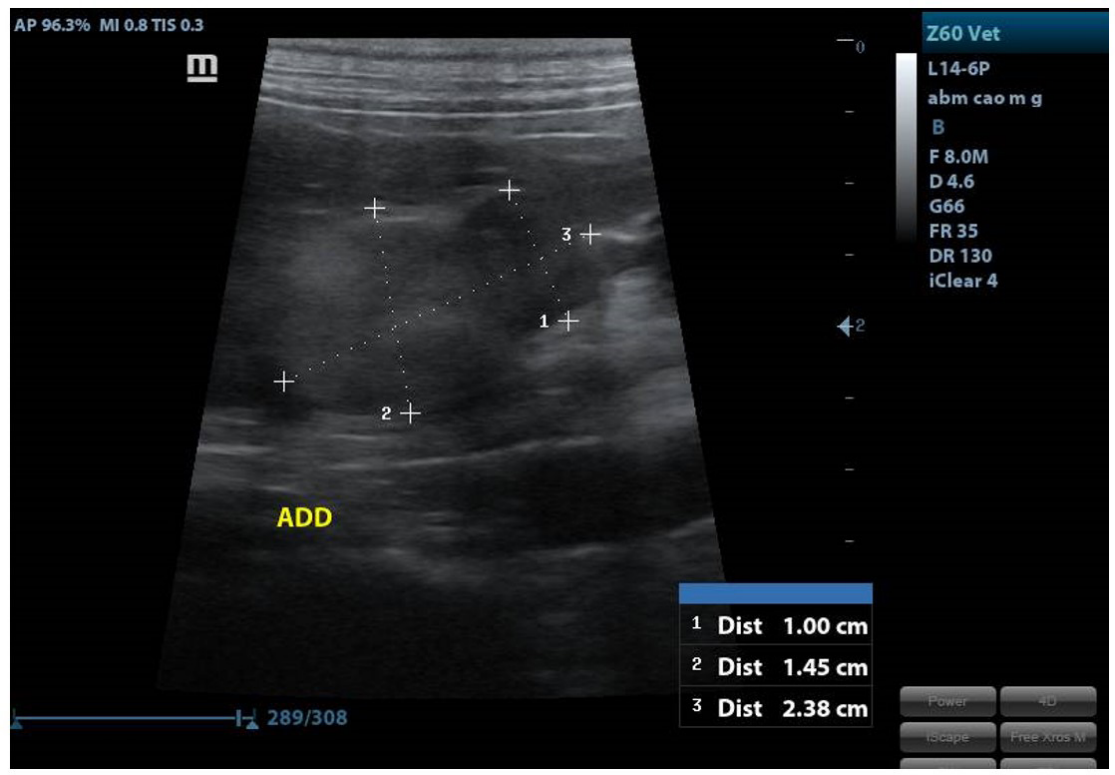
B-mode ultrasound analysis of the right adrenal gland, showing heterogeneity of the parenchyma and increased dimensions.

During the assessment, the dog received a 4/15 on the ALIVE Cushing's Clinical Score. Complementary tests were conducted. Ultrasound showed an enlarged left adrenal gland (0.49 cm caudal pole × 0.71 cm cranial pole × 2.07 cm length) and an enlarged right adrenal gland (1.00 cm caudal pole × 1.45 cm cranial pole × 2.38 cm length) with severely heterogeneous echogenicity and changes suggestive of adrenal neoformation (Figure 1).

The blood count showed no abonormalities, but biochemical analysis showed an elevated alkaline phosphatase level (203 U/L, reference range 10–96 U/L). Based on the ultrasound findings and laboratory tests, hypercortisolism was suspected. A low-dose dexamethasone suppression test was performed, which showed negative results for excessive cortisol production.

**Figure 2:**
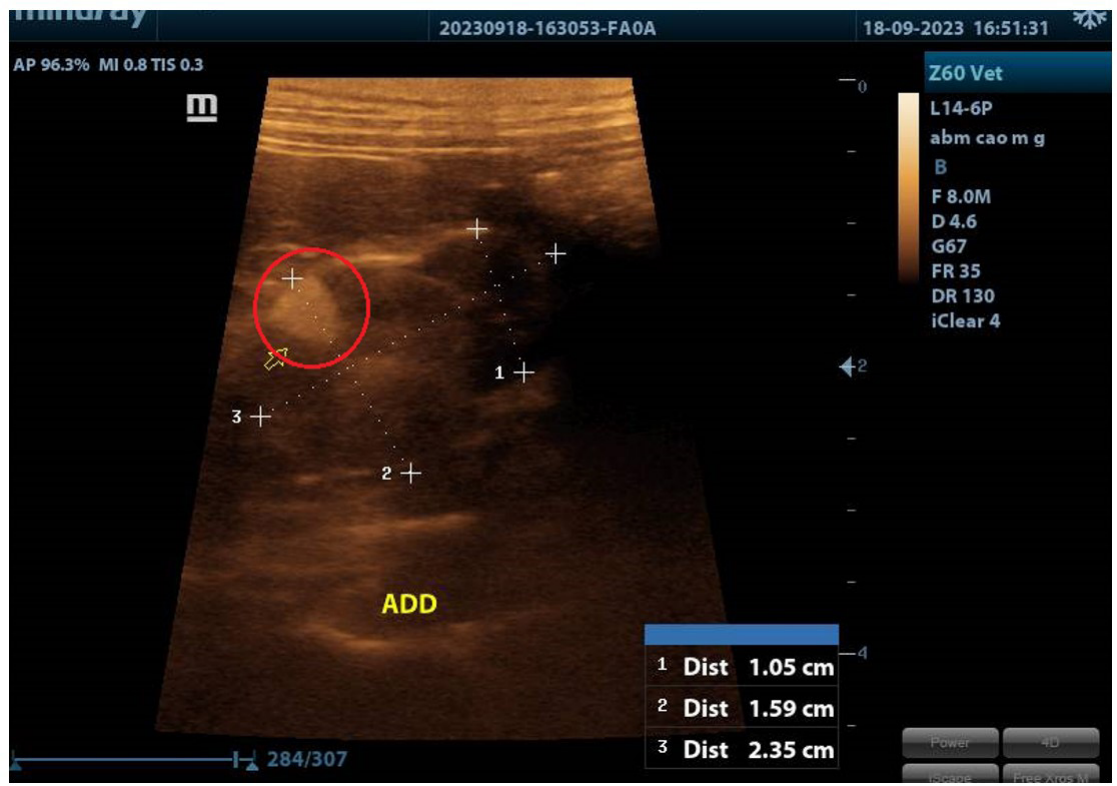
B-mode ultrasound analysis of the right adrenal gland, showing severe parenchymal heterogeneity, mineralization areas in the cranial pole (red circle), and increased dimensions.

After three months, during a follow-up ultrasound, an increase in the dimensions of the left adrenal gland (0.44 cm caudal pole × 0.76 cm cranial pole × 1.79 cm long) and right adrenal gland (1.05 cm caudal pole × 1.59 cm cranial pole × 2.36 cm long) was observed, with worsening parenchymal heterogeneity and the appearance of mineralization areas (Figure 2).

**Figure 3:**
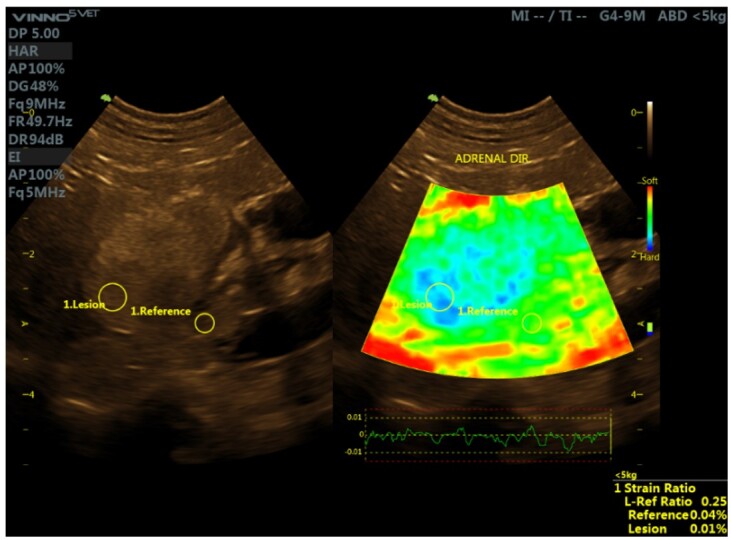
Qualitative and semi-quantitative elastography analysis of the right adrenal.

Together with the B-mode ultrasound evaluation, a qualitative elastography of the right adrenal gland was conducted (Figure 3), in which signs compatible with high tissue stiffness were observed (predominantly bluish tone). Furthermore, in the semi-quantitative evaluation, the gland's stiffness was found to be 75% more rigid than that of the adjacent tissue.

Subsequent laboratory tests showed a continued increase in alkaline phosphatase (AP) levels (358 U/L, reference 10–96 U/L), while other parameters analyzed remained unchanged. The blood count showed slight thrombocytosis at 533,000 mm^3^ (200,000–500,00 mm^3^). A new low-dose dexamethasone suppression test was performed, and the results showed no values compatible with hypercortisolemia.

Considering the ultrasound and elastography findings, together with the progressive enlargement of the right adrenal gland, a CT scan was performed to delimit the size of the gland and assess the vessels and adjacent tissues for the subsequent adrenalectomy.

**Figure 4:**
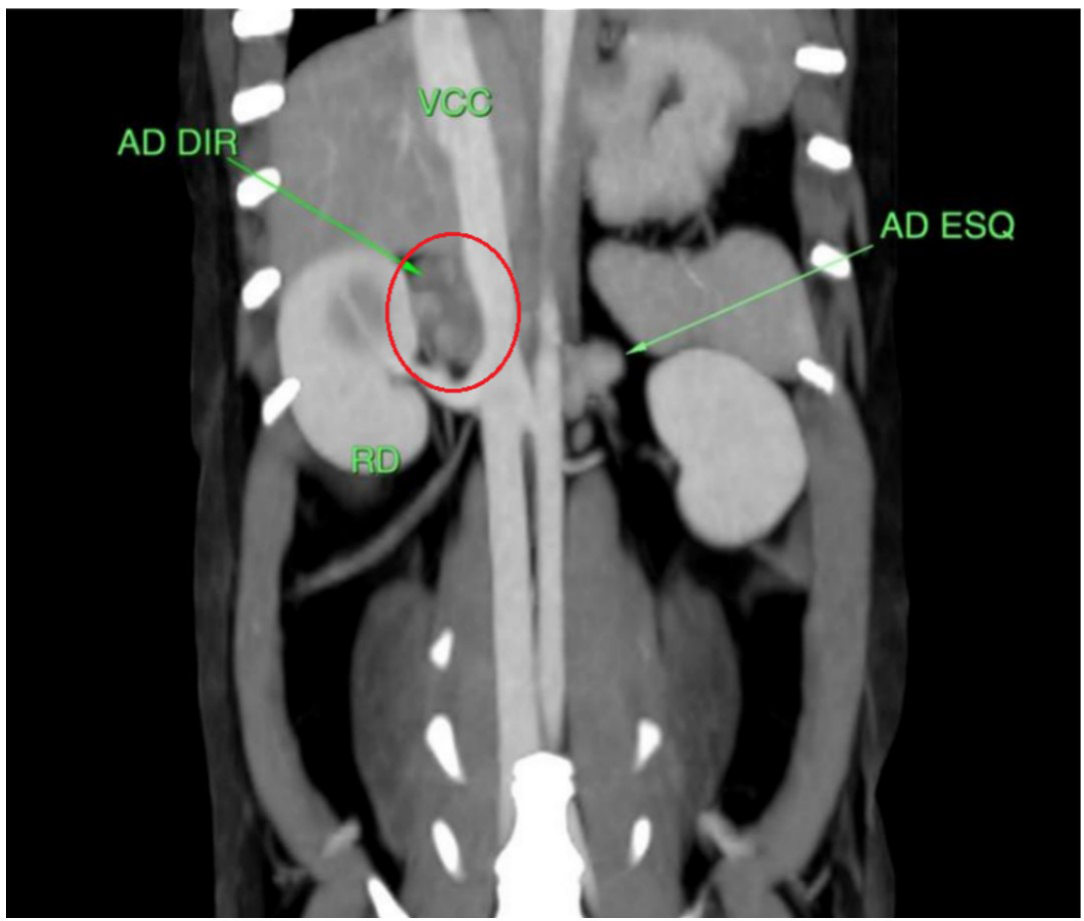
CT image showing ADD (red circle) in close proximity to the caudal vena cava (CCV) and liver. The image was provided by Gamma Vet Centro de Imagem Avançada (Juliana Derenne).

The CT scan (Figure 4) showed an enlarged right adrenal gland, measuring approximately 2.4 cm long, 1.5 cm thick at the cranial pole, and 0.95 cm thick at the caudal pole, with bulging and irregular contours, a slightly heterogeneous appearance with a focal area of fat attenuation in its craniolateral portion and uneven contrast uptake. The lesion intimately contacted and compressed the caudal vena cava and the caudal margin of the caudate process of the hepatic lobe, with probable areas of adherence and no signs of invasion of the vascular lumen.

After performing CT and explaining the case to the guardian, an adrenalectomy was performed. The procedure was uneventful. The patient recovered well with no further complications, and there was no need for corticosteroid or glucocorticoid supplementation. There was significant improvement in skin alterations after surgery. The gland was sent for histopathological and immunohistochemical analyses using the Utrecht score in association with Ki67 morphology.

Histopathological examination revealed malignant tissue characterized by cell groups forming acinar packets and outlines, separated by delicate connective septa, with ample eosinophilic cytoplasm and a medium-sized vesicular nucleus with an obvious nucleolus. The definitive diagnosis was ACC with mitotic figures, capsule invasion, and the absence of necrosis or vascular invasion.

The proliferation marker Ki67 was present in 7% of the neoplastic cells. Histopathological analysis associated with immunohistochemistry identified a Utrecht score of 11, indicating a moderate risk of tumor recurrence.

**Figure 5:**
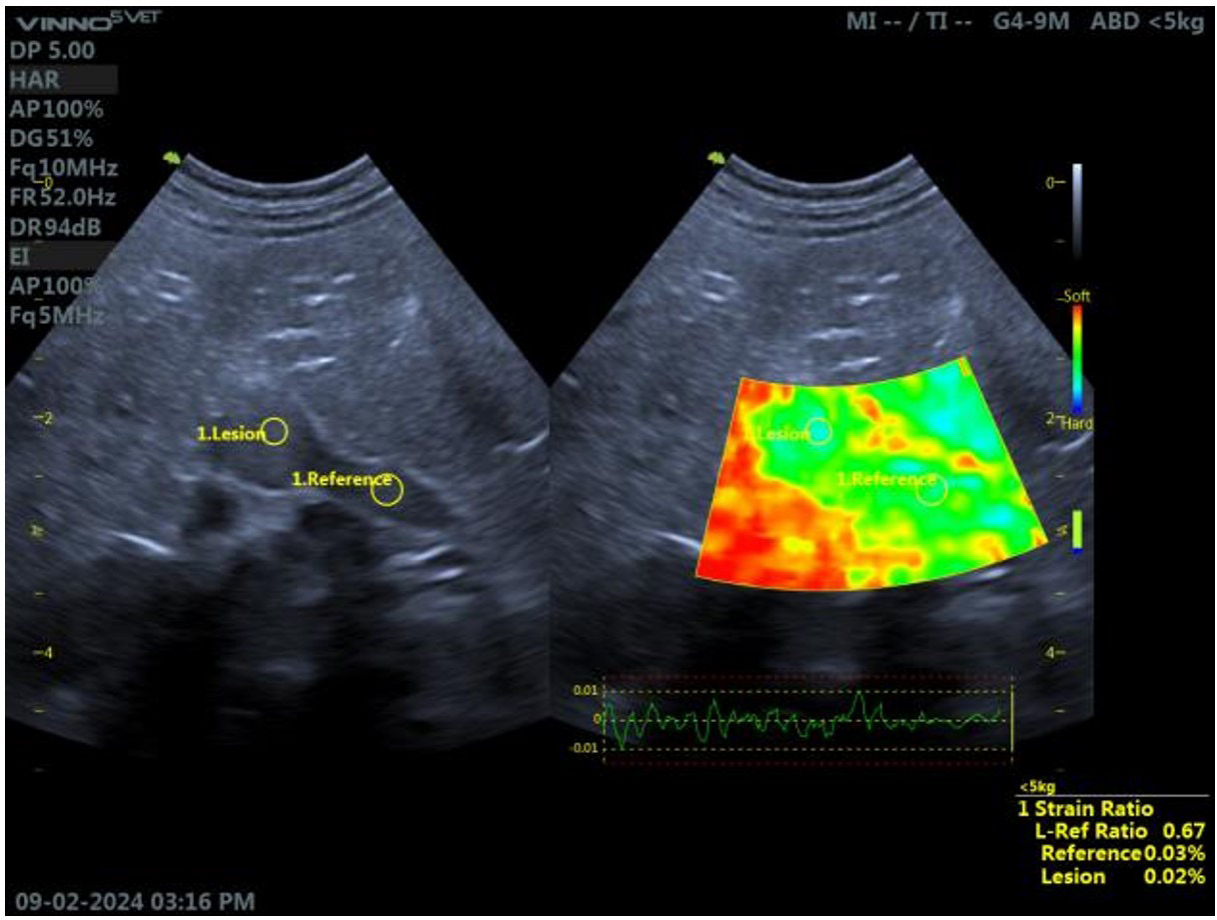
Elastography analysis of the left adrenal gland showing a semi-quantitative comparison between nodular and healthy tissue.

The patient underwent ultrasound follow-up 4 months post-surgery, during which alterations compatible with neoformation in the left adrenal gland were observed. After identifying the lesion in the B-mode, elastography was performed (Figure 5) to obtain more sensitive information about the alteration.

The examination showed the presence of mixed tissue stiffness in the nodule, verified by qualitative elastography, and the semi-quantitative evaluation showed that the nodule tissue was 33% stiffer than the healthy tissue and adjacent mesentery. However, some areas of the nodule were 67% stiffer than the healthy tissue and 75% stiffer than the nodule itself. At the time of writing this manuscript, the patient was monitored for lesions in her left adrenal gland. The patient was clinically stable, with no obvious clinical signs. The guardian was instructed to schedule follow-up appointments with ultrasound and elastography every three months to identify changes early and choose the best therapeutic protocol.

This case report highlights the importance of an integrated approach, from the initial assessment to post-treatment follow-ups, for the effective management of ACC in dogs. This highlights the relevance of imaging techniques in the assessment and prognosis of these clinical conditions.

## Discussion

Dermatological changes are frequently observed in dogs with endocrinopathies (Melián, 2017). Hyperpigmentation, dermatitis, and seborrhea are frequently observed (Bugbee et al., 2023). The main alterations observed in our veterinary patient were skin changes with poor response to treatment, raising the hypothesis of associated endocrinopathy. Biochemical analysis revealed elevated serum (AP) levels, corroborating the changes described by Bennaim et al. (2019). This enzyme can increase when hypercortisolemia occurs due to an increase in the induced cortisol fraction, corroborating the findings of Nelson & Gouto (2014).

The low-dose dexamethasone suppression test is considered the gold standard for identifying hypercortisolemia (Bugbee et al., 2023). Based on the clinical, laboratory, and ultrasound findings, we performed a test to identify hypercortisolemia.

False-negative results can occur with undetected mild hypercortisolemia or in the early stages of endocrinopathy, when the adrenal tumor does not exhibit corticotropin receptors, or through pre-analytical errors (Melián, 2017). The patient in this report did not present values compatible with hypercortisolemia.

A study conducted between 2007 and 2010 showed that 4% of 3,748 dogs had adrenal nodules (Cook et al., 2014). During the ultrasound assessment of the patient in question, an enlargement of the right adrenal gland was observed (1.05 cm caudal pole × 1.59 cm cranial pole × 2.36 cm length) with parenchymal heterogeneity and the presence of mineralization areas. Ultrasound evaluation is extremely important for identifying neoformations.

Elastography provides important information about lesions found in patients with neoformations, as it can assess tissue stiffness (Holdsworth et al., 2014). The data obtained during elastography revealed alterations suggestive of malignancy, as confirmed by histopathological and immunohistochemical examinations. However, it is important to emphasize the need for CT scans for surgical planning and removal of the affected gland (Bennaim et al., 2019).

The staging and prognosis of patients with FAC is determined using histopathological evaluations associated with immunohistochemistry (Sanders et al., 2019). After surgical removal and histopathological examination, hormone-producing ACCs and a Utrecht score of 11 were identified, indicating a high probability of tumor recurrence. Therefore, this report reinforces the findings of Nelson & Gouto (2014), which emphasize the need for imaging tests to detect potential metastasis and assess the contralateral adrenal gland (Nelson & Gouto, 2014). In this report, the patient developed a nodular lesion in the left adrenal gland three months post-surgery. Elastography allowed for a better assessment of the type of lesion, and quarterly follow-ups were indicated.

A study conducted by Massari et al. (2011), which evaluated 25 dogs undergoing adrenalectomy showed an estimated survival expectancy of 953 days, and no signs of tumor recurrence were identified. At the time of writing this report, the patient had survived 355 days, was clinically stable, and was being monitored every three months, although there is evidence of a nodule in the contralateral adrenal gland, whose elastography findings suggest alterations related to areas of fibrosis or neoplastic processes.

## Conclusion

In conclusion, this case report highlights the need for an integrated approach for the effective management of canine ACC. Additionally, this case highlights the importance of continuous monitoring, as new lesions may appear even after initial treatment, requiring early and personalized intervention.
